# Factors associated with psychological wellbeing, is education one of them?

**DOI:** 10.1371/journal.pone.0340820

**Published:** 2026-02-23

**Authors:** Gholamreza Oskrochi, Youssof Oskrochi, Rogheyeh Eskrootchi

**Affiliations:** 1 College of Engineering and Technology, American University of the Middle East, Kuwait; 2 UCL Global Business School for Health, University College London,; 3 Management Information System Department, College of Business Administration, California State University San Marcos, California, United States of America; Ministry of Health, Sri Lanka, SRI LANKA

## Abstract

Many factors, such as health, marital status, social relationships, and job status, influence psychological wellbeing. Previous research has suggested that educational attainment is also a key contributor to a happier life. Utilising data from the Understanding Society survey (2010 -2018), we conducted a comprehensive analysis of the direct and indirect effects of education on psychological wellbeing of 68,283 individuals in the UK. After controlling for relevant variables, we identified a direct association between educational attainment and psychological wellbeing. This aligns with earlier studies examining the link between education and happiness. While some of these studies report significant positive relationships, our results underscore the complexity of factors influencing psychological wellbeing, extending beyond education alone.

## 1. Introduction

Prior to the COVID-19 pandemic, the WHO estimates that approximately 970 million people worldwide lived with a mental disorder, with anxiety and depression being the most prevalent [1]. In the United States, the National Institutes of Health reported that 57.8 million adults had some form of mental illness.

An increasing body of research indicates that psychological wellbeing correlates with positive health outcomes and may also act as a protective factor, diminishing the risk of chronic physical illnesses and contributing to increased longevity. Advocates of this perspective propose that psychological wellbeing merits inclusion in health valuation metrics and should play a role in the allocation of healthcare resources [2].

Psychological wellbeing is linked to numerous physical and social benefits, including improved cognitive function, better health outcomes, and stronger relationships; individuals with higher psychological wellbeing often have better immune function and lower inflammation levels, reducing their risk of chronic diseases [3].

Similarly, education is promoted as a driver for both individual and societal benefits. Individuals stand to gain improved health [4], enhanced self esteem and confidence, better employment prospects, and increased opportunities [5], culminating in overall greater lifetime earnings [6]. Societal benefits, in turn, manifest in heightened trust, diminished political cynicism, and the cultivation of more tolerant attitudes among its members [1].

Despite the evident advantages of education at both levels, the impact of education on psychological wellbeing remains elusive, with existing research yielding conflicting conclusions. Studies range from indicating negative effects [7] to no discernible impact [8, 9] and, conversely, positive effects [10–14].

Recognising this complexity, we aimed to explore the intricate relationship between education and psychological wellbeing, offering a nuanced perspective on this multifaceted interplay.

## 2. Method

### 2.1. Data

This research utilised data from a nationally representative dataset, Understanding Society (The UK Household Longitudinal Study). The study’s observational unit comprises 276,835 valid observations from 68,283 adult individuals who participated in waves 1-8 until the end of 2017. To construct the longitudinal dataset for this study, Understanding Society (US) data were utilised and measured psychological wellbeing along with associated factors using the 12-item General Health Questionnaire (GHQ-12). The GHQ-12 score served as the dependent variable, and its relationship with multiple independent variables such as educational attainment, demographic factors, and financial status was explored.

#### 2.1.1. Data sources.

Understanding Society is a large-scale longitudinal survey covering 40,000 households across the United Kingdom. Launched in 2009 - 2010, it conducts interviews with all eligible household members either face-to-face or via telephone, using Computer-Assisted Interviewing (CAI). The constructed comprehensive dataset encompasses a wealth of information, including educational levels, financial situations, demographic details, and individuals’ psychological wellbeing. The investigation focused on discerning the factors influencing individual psychological wellbeing within this cohort, considering the impact of educational levels, financial situations, and demographic characteristics.

The final model, derived from our analysis, highlighted the most influential variables affecting self-reported psychological wellbeing, as determined by the GHQ-12. These variables included the perception and expectation of current and future financial situations, gender, employment status, age category, marital status, and the number of children at home.

#### 2.1.2. GHQ-12.

The General Health Questionnaire (GHQ) can use as screening tool for detecting psychiatric disorders in the general population. The GHQ comes in several versions (e.g., GHQ-12, GHQ-28, GHQ-30, GHQ-60), with the GHQ-12 being the most preferred due to its brevity, reliability, and ease of use in large-scale studies. Despite its shorter length, it maintains strong psychometric properties while reducing respondent burden, making it highly practical for quick mental health screenings in both clinical and research settings. GHQ-12 has been widely validated across diverse populations, demonstrating sensitivity and specificity comparable to longer versions, ensuring an efficient assessment without significant loss of diagnostic accuracy [15]. Applicable to individuals from adolescence onward (excluding children), the GHQ-12 assesses current psychological wellbeing and is particularly sensitive to short-term fluctuations in mental health.

This self-administered questionnaire focuses on two main domains: the individual’s ability to carry out normal activities, and the experience of new or distressing symptoms. In Understanding Society, the 12 items of the GHQ-12 are included in the self-completion section. Although the overall interview may be conducted face-to-face or by telephone, participants typically complete the GHQ-12 themselves, usually using a paper or digital form without the interviewer reading the items aloud.

#### 2.1.3. Ethical statement.

No ethical approval was required as this study used archived samples from a retrospective, longitudinal study of social, economic, demographic, and health records. The data were fully anonymized and publicly available to researchers.

To access Understanding Society (UK Household Longitudinal Study) data, one need to:

Register with the UK Data Service, Select the dataset (Main Survey), determine the access level and agree to the End User Licence.

For more details, visit: https://www.understandingsociety.ac.uk.

### 2.2. Linear mixed models

Analysing panel (longitudinal) data poses challenges due to temporal correlations in responses and dynamic changes in clustered data variance over time. Neglecting the clustered structure of the data may result in biased estimates and potentially elevated type I error [16,17]. Addressing this issue, Linear Mixed Models (LMM) have been recommended as an effective solution [18–22], accommodating clustered structures and modelling the intricate variance-covariance structure of repeated measurements.

In a similar context, Adams et al [23] applied panel data methods to control for unobserved heterogeneity. Jones and Wildman [24] discussed the non-linearity of the relationship between psychological wellbeing and income, emphasizing the risk of misspecification when neglecting non-linearities. Oskrochi et al [25,26] proposed models employing multivariate linear mixed models with correlated random effects for analysing such panel data. Numerous studies have highlighted the superiority of LMM over General Linear Models (GLM) or Ordinary Least Squares (OLS), as LMM introduces error terms to account for correlations inherent in data clusters (individuals and time), as previously described.

Additionally, LMM facilitates the tracking of psychological wellbeing over time for each individual (treated as random effects in the model) while considering the impact of other time-invariant factors [27]. The literature consistently supports LMM’s superiority over OLS in theoretically modelling various variance-covariance structures to control for potential dependencies due to clustering effects [18]-[22]. An additional argument in Favor of LMM is its more accurate modelling of error terms and control over Type I error rates [28].

The fact that GHQ may exhibit different intercepts between individuals over time (t), LMM enables the treatment of model coefficients as random, incorporating random slope and random intercept models. As the true error structure is typically unknown, comparing criteria is essential for evaluating models with different error structures [27]. A manual procedure will be adopted to select variables and their interactions for the final model, starting with a full model that included all fixed (with interaction between covariates) and random effects. Decisions on retaining variables in the final model and selecting the best variance-covariance structure will be based on comparing differences in deviance assessed by a chi-square test (p < 0.05) with appropriate degrees of freedom [29]. This method aimed to identify the “best” subset of influential factors on GHQ while simultaneously removing redundant variables [30,31].

### 2.3. Dependent or response variable

The measure of psychological wellbeing, derived from the GHQ for each individual, serves as the dependent or response variable in this study. The self-completion questionnaire component of the USS incorporates the GHQ12 as previously described. Respondents use a Likert [32] scale to express recent feelings regarding these elements. The resulting overall score ranges from 12 to 48, where a lower value indicates better psychiatric health. The Mean GHQ was calculated by dividing the total GHQ score by 12, providing an average GHQ for each individual, represented as a quantitative variable between 1 and 4.

**Independent or Risk Factors:**

Various potentially related variables are incorporated into the empirical models to gauge the impact of education and other risk factors. These models control for the financial situation, financial expectations, and other pertinent factors in the reference year, defined as the 12 months preceding the initiation of the interview time. The variables were selected based on their relevance and availability in USS data, ensuring a comprehensive exploration of factors influencing psychological wellbeing. The identified variables are listed below, forming the basis for a robust analysis:

**Scale Variables:**

i) Monthly total personal income and household (HH) income are represented as scale variables. In the parametric mixed models, the logarithm of incomes is employed to facilitate a non-linear relationship between health and incomes [4,33] (Log income & Log HH income).
ii) The log -transformed amount of savings is denoted as Log saving.
iii) The log -transformed amount of rent or mortgage is expressed as Log rent mort.
iv) Age in years at the date of the interview is captured by the variable Age.
v) The number of children in the household is quantified by the variable N Kids.

**Categorical variables**

Gender, divided into 2 categories (Male, Female).

Marital status, divided into 3 categories (Married or Couple; Single; Widowed, Divorced, or Separated).

Expected financial situation a year ahead [Future], divided into 3 categories (Better than now; About the same; Worse than now).

Financial situation now [Fin], divided into 3 categories (Living comfortably or doing alright;

Just about getting by; Finding it difficult).

Problem paying for housing [Prob rent mort], divided into 2 categories (Yes, No).

Highest Educational Qualification, divided into 4 categories (university degree, other higher degree, A level, or at most high school diploma)

Job status, divided into 3 categories (in employment, in training, long term off work)

Owned House, divided into 2 categories (Yes, NO)

Age category, divided into 4 categories (<30, 30–45, 45–65, + 65)

## 3. Analysis and results

The descriptive statistics of the quantitative variables are given in Table 1; and for qualitative variables are given in Table 2.

**Table 1:** Descriptive Statistics of Quantitative Variables.

Variable	Mean	S.D	Min	Max
**GHQ**	1.93	0.47	1.0	4.0
**Income (£/Mon)**	1690.63	1583.76	0.00	27916.33
**House Hold Income (£/Mon)**	3902.91	2696.54	0.00	57841.88
**Saving (£)**	260.96	639.28	0.00	50000.00
**Rent Mortgage (£/Mon)**	452.32	416.94	0.00	4000.00*
**AGE (year)**	42.86	15.33	16	70
NKIDS**	0.57	0.97	0	10

*some outliers (0.15%) are corrected

**NKIDS: is the number of children in the household

For instance, the mean GHQ in this study is 1.93 (sd. 0.47) across 276835 data points.

**Table 2:** Descriptive statistics for categorical socio-demographic variables.

Variable	Categories	(%)
Gender	Male	46%
	Female	54%
Marital status	Married or Couple	52%
	Single	34%
	Widowed, Divorced, Separated	14%
Financial situation, future	Better than now	29%
	About the same	57%
	Worse than now	14%
Financial situation, now	Living comfortably or doing alright	63%
	Just about getting by	26%
	Finding it difficult	11%
Problem paying for housing	Behind with rent or mortgage?	
	No	91%
	Yes	9%
Highest educational Qualification	Degree.	25%
	Other Higher degree	12%
	A level or equal	23%
	GCSE or equal	22%
	Other qual	8%
	No qual	10%
Job status	Self-Employed, retired, Employed	75%
	Student and training	8%
	Unemployed or looking after family	12%
	Long Term Sick	4%
	Doing something else	1%
House Owned	Yes	68%
	No	32%
Age category	<30 years	24%
	30–45	27%
	45–65	36%
	> 65	13%

To investigate the association between the factors and GHQ, pairwise correlation analyses were conducted, the results are presented in Table 3.

**Table 3:** Pairwise correlation matrix for all variables.

	Log income	Log HH income	Log save	Log rent	age	NKIDS
**GHQ**	−.036**	−.109**	−.074**	−.002	.001	.009
**Log income**		.317**	.152**	.066**	.285**	.121**
**Log HH income**			.144**	.155**	−.082**	.066**
**Log save**				.022**	.023**	−.035**
**Log rent**					−.316**	.204**
**age**						−.161**

**. Correlation is significant at the 0.01 level (2-tailed).

All factors exhibited significant negative correlations with GHQ, except Number of Children (NKIDS) and age, which demonstrated a significant positive correlation at the 5% level. Age exhibited the least correlation with GHQ. These findings are summarized in Table 3 for a comprehensive understanding of the relationships.

Various variance-covariance structures were tested to assess the error covariance structure of the longitudinal data, allowing the intercept to vary across participants. The final validated model, employing random effect mixed models, underwent assessment using standard diagnostic tools. All mixed effects models were fitted using the Random effects and population average Maximum Likelihood linear regression models in STATA ver. 16.

The general form of mixed effects model used is


$${GHQ}_{ij}={(\beta }_{oo}+\beta_{\text{10}}t_{ij}+\beta_{\text{20}}{t^{\text{2}}}_{ij})+\left(b_{\text{0}i}+b_{\text{1}i}t_{ij}\right)+\left(\beta_{\text{2}}AGE+\beta_{\text{3}}AGE^{\text{2}}\right)+\ldots +\beta_{k}X_{k}+\varepsilon_{ij}$$

where ${GHQ}_{ij}$ is for individual i, at time (year) j, $\beta_{oo}$ is the overall GHQ mean and $b_{\text{0}i}$ is the individual deviation from this mean, $\left[b_{\text{0}i}\sim N\left(\text{0},\sigma_{\text{0}}^{\text{2}}\right)\right]$. Similarly, $\beta_{\text{1}\text{0}}$ is the mean growth/decline rate in GHQ. $b_{\text{1}i}$ is the random (slop) individual psychological wellbeing growth/decline mean $(b_{\text{1}i}\sim N\left(\text{0},\sigma_{\text{1}}^{\text{2}}\right)$. $X_{k}$ *(for k = 4,5 ….)* are all other explanatory variables (covariates) including highest education qualification ever achieved with their associated coefficient $\beta_{k}$ (for k = 4, 5, ….). Conditional on given random effects, $\in_{ij}$ are supposed to be independent random errors for each individual i and time j, $\in_{ij}\sim N\left(\text{0},\sigma_{\varepsilon }^{\text{2}}\right)$.

To test a nonlinear growth trajectory across time, other higher-order polynomial trends (i.e., quadratic) was also tested to investigate if it can improve model fit.

The covariates included in the model to assess their impact on self-reported psychological wellbeing (GHQ) encompass log-transformed individual income, log-transformed household income, log-transformed savings, log-transformed rent or mortgage payments, Gender, marital status, number of children in the household, job status, expected future financial situation (Finfut), current financial situation (Finnow), problems with rent or mortgage, highest educational qualification (Hiqual), home ownership, age categories, and relevant interactions. The associated coefficients are denoted as β_4_, β_5_,..., β_k_.

The model accounts for interactions between time and potential predictors, as well as confounding factors. Additionally, interactions between other covariates, such as Gender and Marital Status or Gender and household income, including Gender and Higher degree, were incorporated to examine whether their effects varied across responses over time.

To determine the most appropriate covariance structure, various autocorrelation structures, such as AR1 or moving average autocorrelation, were considered. Model selection was guided by changes in deviance. A meticulous visual inspection of residual plots did not reveal any substantial deviations from distributional assumptions, affirming the suitability of the fitted Linear Mixed Model for the analysis [34].

### 3.1 Univariate analysis

The analysis was first performed univariately to assess the effect of each risk factor (covariate) independently. The results are summarised in Table 4.

**Table 4:** Univariate analysis of all covariates.

Covariate	Coefficient	Std Err	Z	P-value
Time (wave)	−0.000	0.000	−1.470	0.140
Gender (male)	−0.100	0.003	−34.130	0.000
Age	0.000	0.000	5.010	0.000
Higher deg.	−0.100	0.005	−19.190	0.000
A level	−0.074	0.005	−15.050	0.000
GCSE	−0.058	0.005	−11.870	0.000
N Kids	0.004	0.001	3.520	0.000
Log income	−0.005	0.000	−10.160	0.000
Self emp, Employed	−0.148	0.003	−52.650	0.000
In training student	−0.146	0.004	−35.540	0.000
Long-term sick	0.288	0.005	52.540	0.000
Financially Comfortable	−0.484	0.005	−102.440	0.000
Financially Good	−0.431	0.005	−94.570	0.000
Financially OK	−0.333	0.005	−74.030	0.000
Financially diff	−0.184	0.005	−38.090	0.000
Future better	−0.089	0.003	−34.390	0.000
Future same	−0.082	0.002	−36.570	0.000
Married	−0.024	0.003	−8.650	0.000
Separated	0.112	0.007	17.010	0.000
Divorced	0.060	0.004	13.350	0.000
Widow	0.065	0.008	8.200	0.000
House Owned	−0.073	0.003	−27.480	0.000
Log HH income	−0.033	0.001	−30.750	0.000
Age	0.015	0.001	21.530	0.000
Age²	−0.000	0.000	−23.130	0.000
Age < 30	0.010	0.005	1.790	0.074
30 ≤ Age < 45	−0.010	0.004	−2.680	0.007
45 ≤ Age < 65	0.008	0.003	2.580	0.010
Problem rent/mortgage	0.061	0.003	21.550	0.000
Log saving	−0.006	0.000	−16.310	0.000
Log rent/ mortgage	−0.001	0.000	−1.620	0.105

### 3.2 Multivariate analysis

The analysis performed using multivariate regression with LMM, to assess the effect of each risk factor (covariate) while controlling for all other factors. The results are summarised in Table 5.

**Table 5:** Multivariate analysis with LMM.

Variable	Coefficient	Std. Err.	z	P Value	95% CI.
Time	0.002	0.001	1.580	0.115	−0.001–0.005
Gender	−0.053	0.017	−3.090	0.002	−0.086 – −0.019
N Kids	−0.015	0.001	−10.300	0.000	−0.017 – −0.012
Log income	0.004	0.001	7.130	0.000	0.003–0.005
S/Employed	−0.102	0.003	−32.620	0.000	−0.108 – −0.096
Student training	−0.065	0.005	−14.100	0.000	−0.074 – −0.056
Long term sick	0.261	0.006	47.150	0.000	0.250–0.272
Financially Comfort	−0.427	0.005	−86.070	0.000	−0.437 – −0.418
Financially Good	−0.382	0.005	−80.240	0.000	−0.391 – −0.372
Financially OK	−0.294	0.005	−63.440	0.000	−0.304 – −0.285
Financially Diff	−0.160	0.005	−32.280	0.000	−0.169 – −0.150
Future Better	−0.088	0.003	−34.190	0.000	−0.093 – −0.083
Future Same	−0.066	0.002	−29.520	0.000	−0.070 – −0.061
Married	−0.041	0.004	−10.330	0.000	−0.049 – −0.033
Separated	0.036	0.007	5.330	0.000	0.022–0.049
Divorced	0.001	0.005	0.240	0.812	−0.008–0.010
Widow	0.024	0.008	3.080	0.002	0.009–0.040
Degree	−0.037	0.006	−6.410	0.000	−0.048 – −0.025
A level	−0.010	0.005	−2.090	0.037	−0.019 – −0.001
GCSE	−0.014	0.005	−2.950	0.003	−0.023 – −0.005
House owned	−0.005	0.003	−1.740	0.082	−0.010–0.001
Log HH income	−0.002	0.002	−1.210	0.225	−0.005–0.001
Age < 30	−0.003	0.005	−0.520	0.603	−0.013–0.008
30 ≤ Age < 45	−0.007	0.004	−2.050	0.040	−0.014 – −0.000
45 ≤ Age < 65	−0.007	0.003	−2.150	0.032	−0.013 – −0.001
Prob rent/mort	0.016	0.003	5.490	0.000	0.010–0.021
Log saving	−0.001	0.000	−3.280	0.001	−0.002 – −0.000
Log rent/mort	0.000	0.000	0.660	0.508	−0.000–0.001
Gen/Mar interaction	0.036	0.005	7.450	0.000	0.026–0.045
Gen/Deg interaction	0.043	0.006	7.370	0.000	0.032–0.055
Gen/HH in interaction	−0.008	0.002	−3.730	0.000	−0.012 – −0.004
Time²	0.001	0.000	17.840	0.000	0.001–0.001
Age²	−0.000	0.000	−15.780	0.000	−0.000 – −0.000
Age	0.012	0.001	14.600	0.000	0.010–0.013
Constant	2.243	0.022	102.240	0.000	2.200–2.286

The optimal model (Table 5) was chosen by retaining the significant factors through the implementation of the most suitable variance covariance structure. In the selection process, a preference was given to smaller deviance values (−2 times the logarithm of likelihood) in relation to their respective degrees of freedom.

Notably, the estimates of the intercept and quadratic slope (Time^2^) in the model proved to be both significant and realistic, with p-values of less than 0.001. The analysis strongly suggests that the random slope model is ill-suited for this dataset, as time exhibits no discernible linear effect on the growth or decline of GHQ. Therefore, for the sake of simplicity, the random intercept model over time was applied to the data.

Further examination indicates that all factors listed in the table are significantly linked to self-reported psychological wellbeing, except for the absence of a distinction between divorced and single individuals. Additionally, owning a residential home, household income, and the logarithmic amount of rent or mortgage are not directly associated with self-reported psychological wellbeing.

Conducting a likelihood ratio test to compare the final fitted random effect model with a conventional model revealed a noteworthy improvement, with a gain of over 57000 in deviance (−2log-likelihood) for 2 degrees of freedom, yielding a p-value of <0.0001. Furthermore, the reduction in deviance for the random effect model, considering all covariates, compared to the same models without covariates, is 22971.56 for 34 degrees of freedom, returning a p-value of less than 0.00001.

The model estimates (Table 6) reveal an intercept of 2.243 (SE = 0.022) and a slope (time) of 0.002 (SE = 0.001). The intercept proves to be statistically significant (p < 0.001), indicating a meaningful starting point. However, the slope lacks significance, suggesting an absence of linear growth or decline over time.

**Table 6:** Model’s estimates.

Variables (Fixed effects)	Coefficients (Standard Error)
Intercept	2.215 (0.006)***
Time (Year)	2.722 (0.707)***
Time²	−5.322 (0.470)***
Financially Good	−0.296 (0.003)***
Financially Surviving	−0.194 (0.003)***
Financially Difficult	Reference category
Future Better	−0.023 (0.002)***
Future Worse	0.057 (0.002)***
Future Same	Reference category
AGE	−4.401 (0.855)***
AGE²	−2.702 (0.646)***
AGE³	11.749 (0.608)***
NKIDS	−0.012 (0.002)***
Single	0.017 (0.004)***
Wido/Div/Sep	0.060 (0.004)***
Married or couple	Reference category
GCSE	−0.030 (0.005)***
HND, NVQ	−0.063 (0.006)***
Technical	−0.048 (0.005)***
No Qualification	Reference category
Long term sick	0.277 (0.005)***
Temporary Job	0.099 (0.004)***
Stable Job	Reference category
**Random Components**	**Standard Deviation**
Intercept	0.260***
Slope	0.011***
Residuals	0.340
AIC	178481.400
R²	0.592

**Fig 1:**
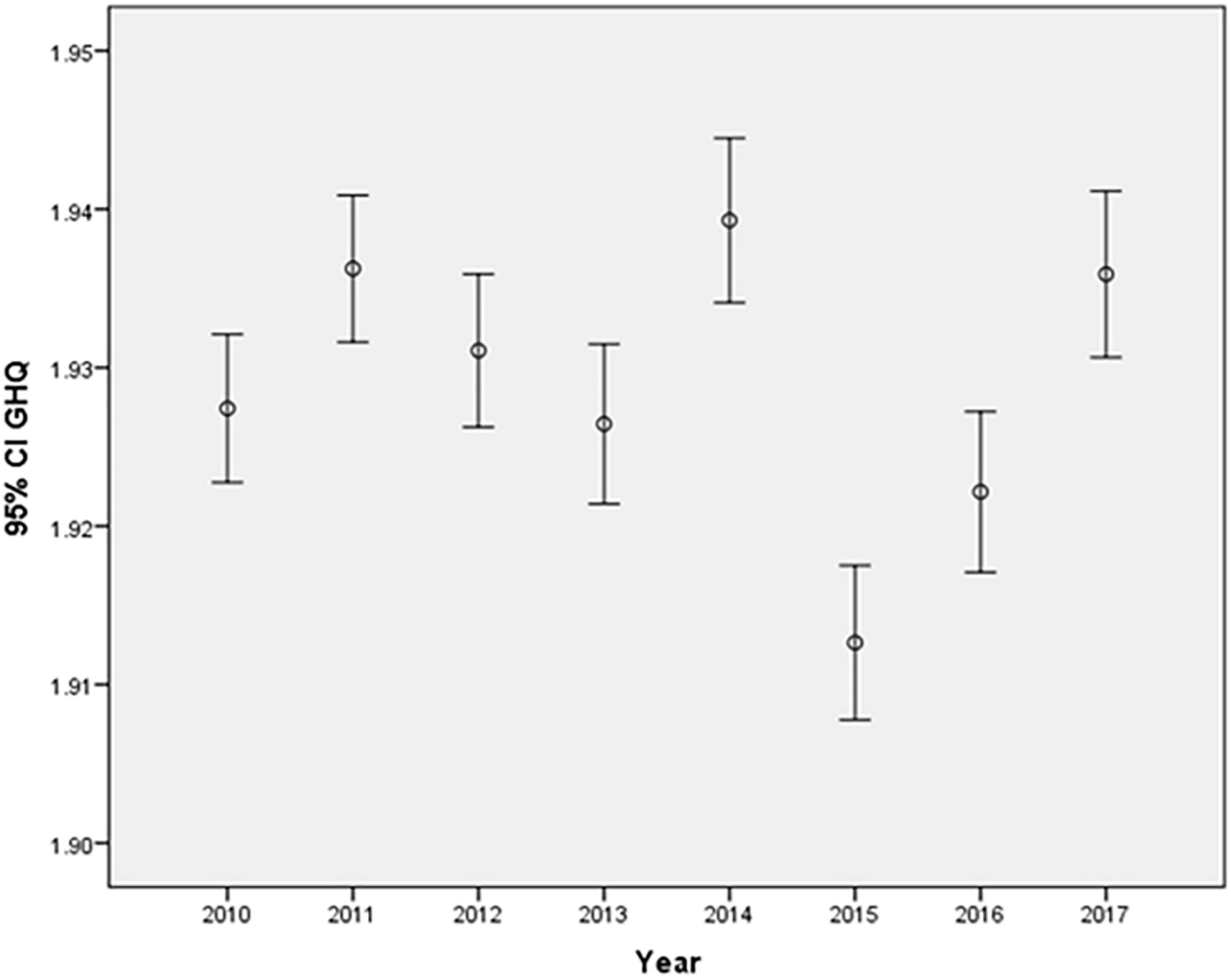
Time effect.

The inclusion of time squared in the model yields a significant contribution (p < 0.001), signifying a nonlinear effect of time on the GHQ. The positive and significant effect of the quadratic time implies a growth in GHQ scores, with this growth intensifying in later periods (Time effect – Fig. 1).

## 4. Interpretation of the results

### 4.1. Univariate analysis result

There was no significant change in average GHQ over time (*p* = 0.140). However, the following are all statistically significant (with p < 0.05)

- Males generally reported better psychological wellbeing (lower GHQ scores).
- Older age was associated with lower confidence in psychological wellbeing.
- Higher educational qualifications were linked to better reported mental health compared to having no qualifications.
- A greater number of children in the household was associated with lower confidence in psychological wellbeing.
- Higher income was linked to greater confidence in psychological wellbeing.
- Being employed or in training was associated with higher confidence in psychological wellbeing compared to being unemployed.
- Long term illness was linked to lower confidence in psychological wellbeing, even compared to unemployment.
- Perceived current financial situation was directly related to psychological wellbeing—greater confidence in finances correlated with higher psychological wellbeing.
- Perceived future financial security was also positively associated with psychological wellbeing - the better the outlook, the greater the confidence in psychological wellbeing.
- Compared to single individuals, married individuals reported fewer psychological problems, while separated, widowed, and divorced individuals reported more problems.
- Homeownership was associated with greater confidence in psychological wellbeing.
- Higher household income was linked to increased confidence in psychological wellbeing.
- Psychological distress generally increased with age; however, the quadratic age term (age²) indicated that individuals over 65 experienced fewer problems. The age group reporting the best GHQ scores was 30 - 45, while the worst scores were observed in those aged 45–65.

The Mean GHQ, on a scale of 1 - 4, where lower values denote a better perception of health, experienced a dip in 2015 and 2016. However, a comprehensive analysis suggests that this decline may be attributed to other factors rather than a direct association with time.

### 4.2. Multivariate analysis result

**Fig 2:**
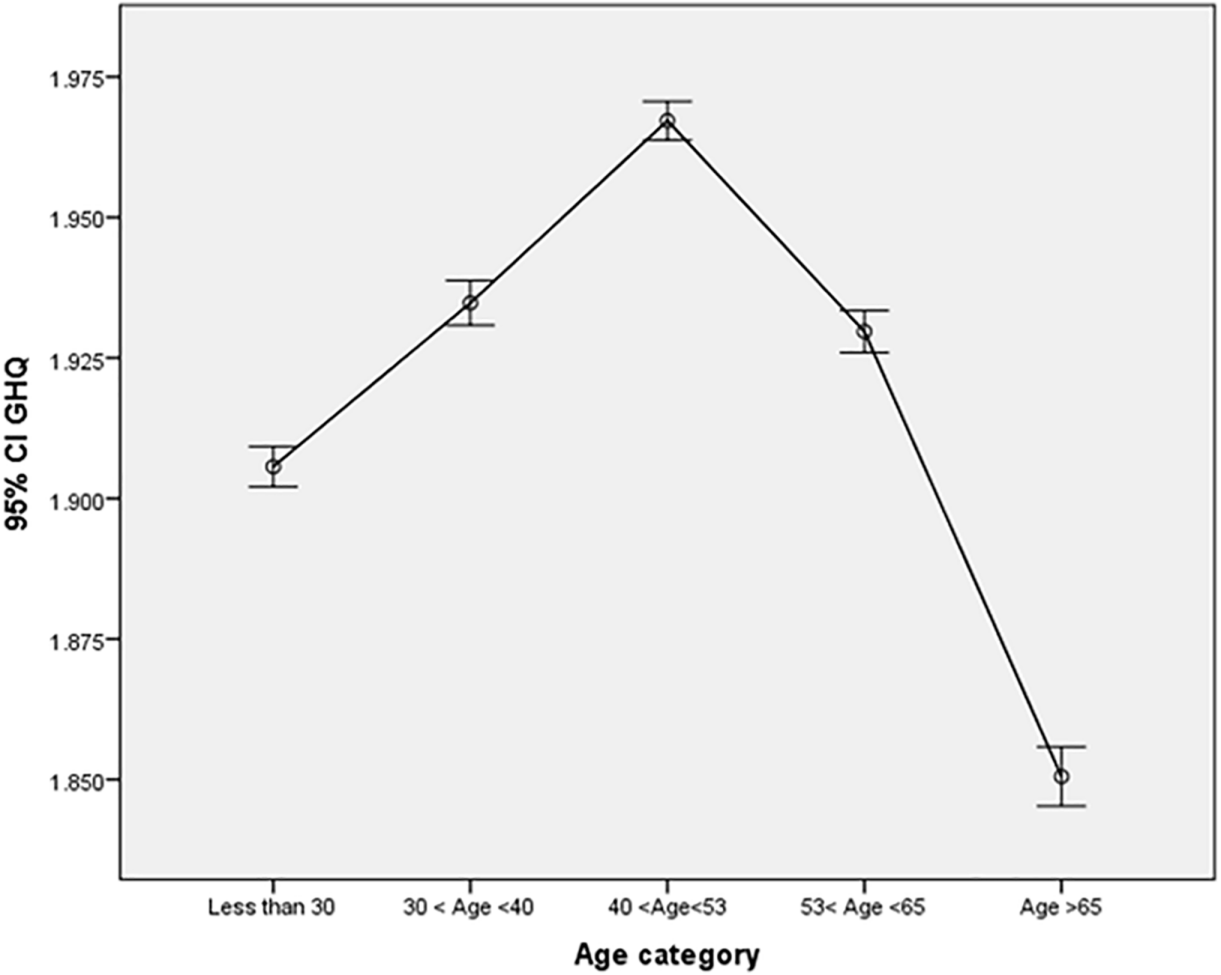
Age effect.

Again, there was no significant change in average GHQ over time (p = 0.115). Concerning demographic covariates, males exhibited consistently lower (improved) GHQ score averages than females (β = −0.053, p = 0.002). The influence of age was identified as nonlinear and significant, revealing an increase (deterioration) in GHQ scores until around the age of 50, followed by a subsequent decline (Age effect – Fig 2).

Furthermore, the number of children displayed a noteworthy impact, correlating with a reduction (improvement) in GHQ scores per child (β = −0.015, p < 0.001).

Marital status (MASTAT) played a significant role in our analysis. Married individuals exhibited a notably lower (improved) GHQ score (β = −0.041, p < 0.001) compared to their single counterparts (reference category). Conversely, widowed and separated individuals showed significantly higher GHQ scores than singles (β = 0.024 and 0.036 respectively, with p < 0.001).

**Fig 3:**
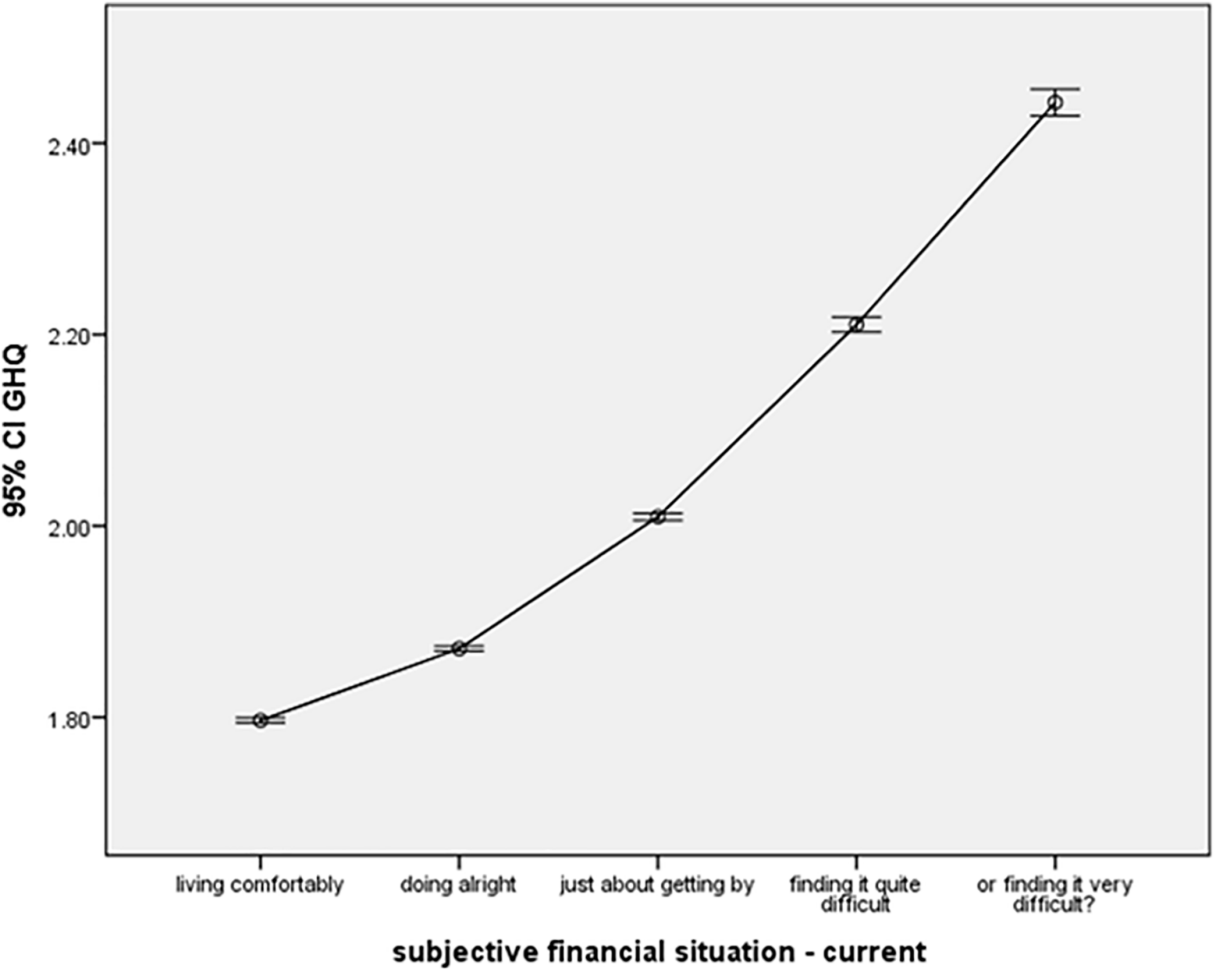
Financial situation effect.

Our modelling further indicates a strong correlation between current financial situation (Fin.) and psychological health (Fin. effect – Fig 3). The most substantial and significant decrease (improvement) in GHQ average score was observed in those “living comfortably” (β = −0.427, p < 0.001), followed by those “doing good” (β = −0.382, p < 0.001), “doing OK” (β = −0.294, p < 0.001), and “finding it difficult” (β = −0.160, p < 0.001) when compared to the reference category of those “finding it very difficult.” The accompanying graph illustrates the average GHQ scores across different levels of current financial situations.

**Fig 4:**
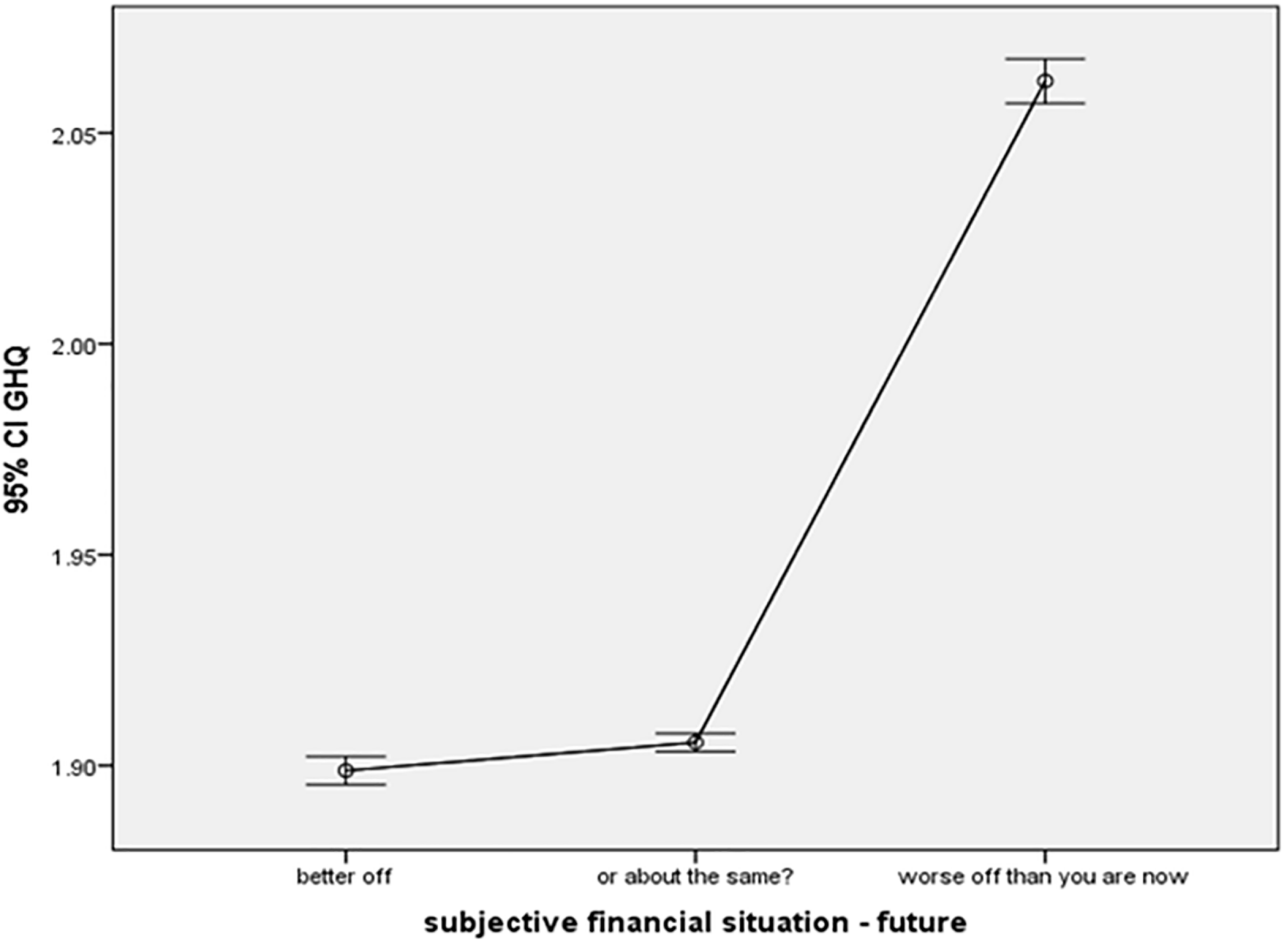
Future Financial situation effect.

Financial expectations for the year ahead (Fut.) reveal a parallel trend (Fut. effect – Fig 4). Individuals anticipating a better financial year ahead exhibited significantly better GHQ average scores (β = −0.088, p < 0.001) compared to those expecting no change (reference category). Likewise, individuals expecting the same financial status also showed improved GHQ scores (β = −0.066, p < 0.001) when compared to those anticipating a negative financial situation next year. The accompanying graph visually represents the average GHQ scores across various levels of future financial situation.

Income, though not found to be statistically significant, exhibited substantial variability explained mainly by job status and financial situations. For the sake of simplicity and model parsimony, income was omitted, while job status and financial situations were retained using the previously explained methodology.

However, log income emerged as statistically significant (β = 0.004, p < 0.001) and displayed a negative association with GHQ when controlling for all other factors. This observation may suggest that individuals in higher-income jobs experience heightened stress when other variables are considered. However, log household income (HH) exhibited a positive association with improved psychological wellbeing, but not significantly (p = 0.225).

**Fig 5:**
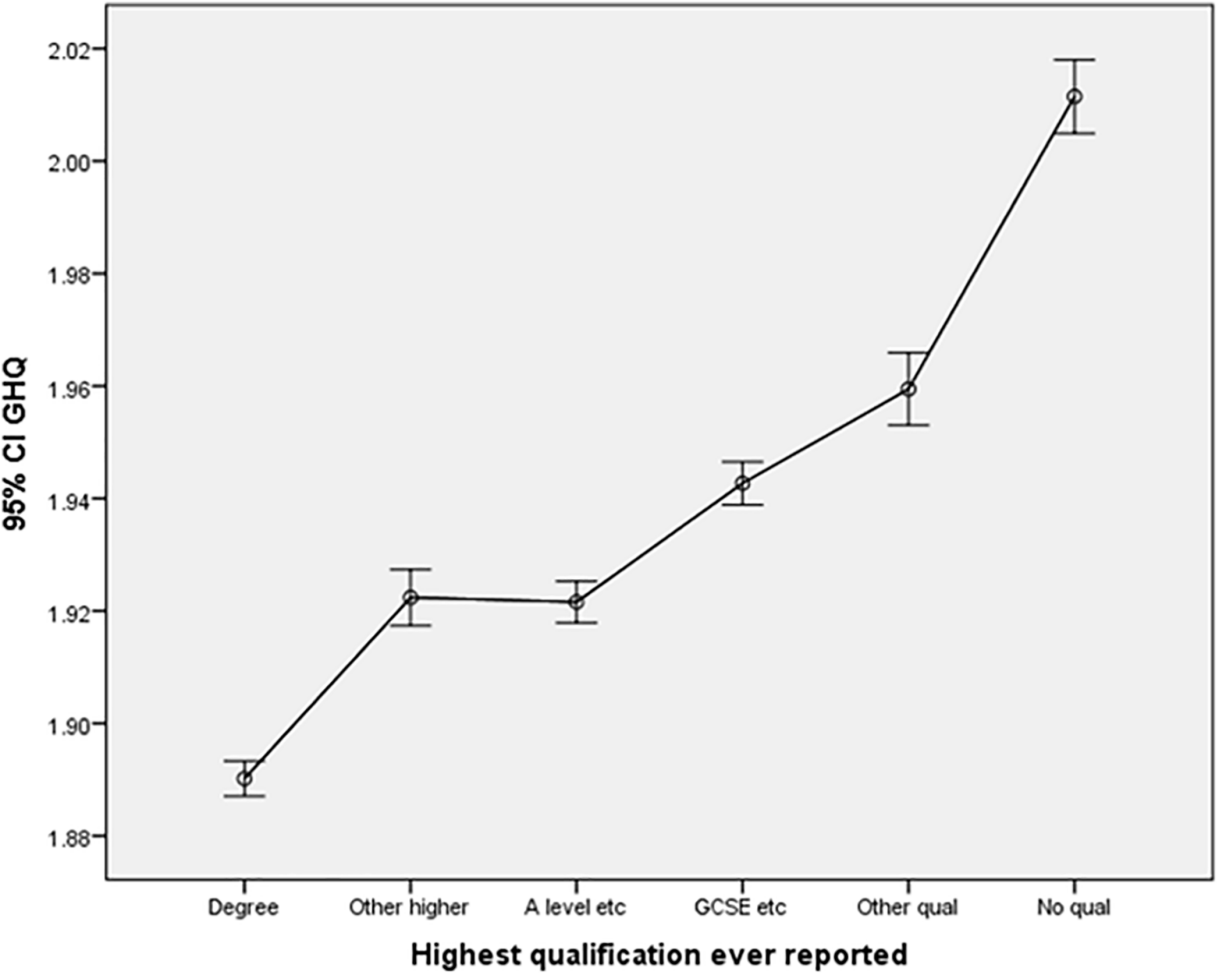
Educational qualification effect.

Regarding educational attainment, individuals with a university degree (β = −0.037, p < 0.001), A-level qualification (β = −0.01, p < 0.05), and GCSE qualification (β = −0.014, p < 0.005) demonstrated significantly lower GHQ scores compared to those with no qualifications. The accompanying graph (Educational qualification effect – Fig 5) visually depicts the average GHQ scores across different levels of educational qualifications.

**Fig 6:**
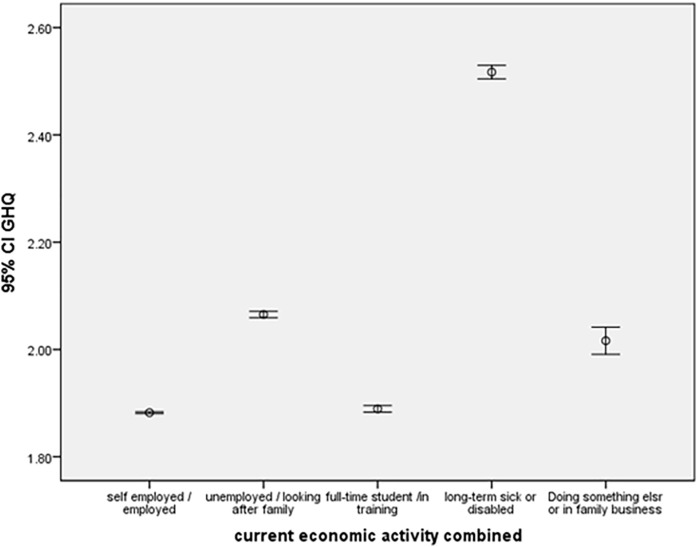
Employment effect.

Finally, job status was also significantly influential on GHQ score (Employment effect – Fig 6). Those deemed long term sick (β = 0.261) have significantly lower psychological health compared to unemployed individuals (reference category). Employed and self-employed individuals significantly have better psychological health compared to unemployed individuals, followed by those individuals who are in training. The following graph shows the average GHQ for different level of Job status.

The challenge of meeting rent or mortgage payments is significantly linked to an increase in GHQ, indicating a correlation with poorer health. On the flip side, possessing savings is associated with better psychological health, reflecting a positive impact.

In exploring interaction terms, noteworthy findings emerge. Gender exhibits interactions with marriage (Gen/Mar int.), higher educational degrees (Gen/Deg int.), and household income (Gen/HH in int.). While being male and married independently contribute to reduced GHQ and improved psychological health, the effects are not simply additive, necessitating a correction for the interaction of married and male statuses. Similarly, the same correction is required for the interaction of males and higher educational degrees. Interestingly, males in high income households experience less pressure and demonstrate a significant reduction in GHQ. These nuanced interactions shed light on the complex interplay of various factors influencing psychological health.

## 5. Discussion

A significant relationship is observed between educational attainment and psychological wellbeing. Higher levels of education are positively associated with improved psychological wellbeing; however, our findings indicate that psychological wellbeing is influenced by multiple factors and cannot be attributed solely to education. Protective factors associated with lower GHQ scores and the promotion of psychological wellbeing include male gender, marital status, age, presence of children, employment status (either employed or self-employed), current and perceived future financial expectations, challenges with paying rent or mortgage, household income, and individual income.

Our findings align with established associations between financial status, education level, male gender, and mental wellbeing scores, corroborated by national-level surveys [35,36].

Our analysis however introduces a further nuanced perspective, highlighting that the most influential factors for psychological wellbeing are current and future financial expectations, followed by education level. Notably, higher individual income surprisingly does not correlate with improved psychological wellbeing, whereas higher household income is associated with enhanced psychological wellbeing.

This discrepancy may be rationalised by the definitions and connotations of income versus financial status. Income, being an absolute measurement, is subject to “hard” adjustments such as local taxes and deductions - factors beyond individual control. In contrast, financial status can be viewed as a more holistic assessment with greater individual influence. When evaluating their financial status, individuals consider not only income but also additional factors like debt levels, savings, expectations for investments, spending habits, and the earning potential of their immediate family or partner. Moreover, financial status encompasses “soft” influences like the perceived standard of living, future expenditure plans, and the ability to afford desired comforts, such as living comfortably.

Differences in life experiences influence subjective perceptions of financial status. Individuals who have endured and surmounted financial hardships may interpret their current financial situation more positively [37]. Therefore, those with high initial expectations might feel deserving of more. Additionally, an individual’s perception of the importance of financial success as an indicator of overall success cannot be overlooked. Strong aspirations for financial success, to the point of overshadowing other life goals, have been linked to various psychological issues in previous research [38].

The implications of our analysis challenge the conventional approach of solely considering income when analysing financial effects on individuals. Treating income in isolation is deemed a crude measure at best and potentially erroneous at worst. Human perception of the world is intricately shaped by future expectations coloured by past experiences. Therefore, adopting a more holistic variable that incorporates these elements proves to be a more appropriate method for comprehensively analysing the impact of financial factors on psychological wellbeing.

Our findings align with previous research highlighting the positive impact of marriage or having a partner on psychological wellbeing. Wilson and Oswald’s longitudinal review [39] emphasized that marriage reduces the risk of psychological illness, promoting happiness and overall health. Intriguingly, they suggested that men may derive more benefits from marriage than women, providing a potential explanation for the observed gender differences in psychological wellbeing scores.

Additionally, we highlight the positive impact of having children on psychological wellbeing. While recent evidence in this area remains somewhat mixed, with a slight inclination toward a positive effect, the consensus suggests that the impact of children depends on life stage. More importantly, it is not just the number of children that matters, but the quality of the parent-child relationship, which plays a crucial role in shaping parental psychological wellbeing [40]. Child age emerges as a crucial factor, with parents of younger children reporting generally better psychological wellbeing scores [41]. Various explanations range from the increasing autonomy of adolescent children [42] to changes in parent-child dynamics, including less time spent with parents and questioning of parental rules and practices [43].

Moreover, our analysis underscores the role of job stability and security in improving mental health and overall wellbeing, consistent with academic and policy advice [44]. A meta-analysis [45] highlights the significant impact of job insecurity on both mental and physical health, emphasizing the anticipation of undesirable events, such as unemployment, as a source of psychological strain in line with stress theory.

Contrary to De Witte’s findings [46], suggesting that job insecurity was a significant risk factor for psychological wellbeing in men but not women, our analysis challenges this gender-specific conclusion. We indicate that job insecurity affects both genders, emphasizing the importance of gender equality considerations in understanding the impact of job insecurity on psychological wellbeing.

Furthermore, treating job security and insecurity as binary states and applying their effects on psychological wellbeing as discrete events is deemed inappropriate. Ferrie et al.‘s review [47] of chronic job insecurity demonstrates that the psychological issues stemming from periods of job insecurity persist even after job security has been restored. This suggests that the anxiety and stress associated with job insecurity are enduring, and individuals who have experienced job insecurity may always carry an expectation of experiencing it again at some level.

The impact of education on psychological wellbeing also varies based on cultural factors. In some societies, educational achievement may contribute more significantly to psychological wellbeing. For instance, studies in East Asia [48], show that education is deeply intertwined with collective social expectations, which can influence both individual and communal psychological wellbeing.

According to UNICEF [49] education is a key determinant in achieving sustainable health outcomes, especially in low-income countries. The report emphasises that education is a fundamental driver of child psychological wellbeing globally, as educated mothers, for example, tend to have healthier children and are better able to advocate for their families’ health needs.

The WHO [50] stresses that education is linked to lower rates of mental disorders in developed nations, but the opposite may be true in areas with inadequate mental health services.

### 5.1. Strengths and Limitations

This model’s strength lies in its ability to accommodate both fixed and random effects for both the intercept and the slope, providing a robust framework for analysing the dynamics of longitudinal data.

However, it’s important to note a limitation in this analysis - it does not track a specific cohort over time to explore the evolving impact within the same group.

While the Linear Mixed Model proves effective for capturing general trends, a cohort-focused investigation could offer deeper insights into the nuances of change within a particular group over the study period.

## 6. Conclusion

A direct association between educational attainment and psychological wellbeing is identified. This aligns with earlier studies examining the link between education and happiness. While some of these studies report significant positive relationships, our results underscore the complexity of factors influencing psychological wellbeing, extending beyond education alone.

This paper introduces several novel contributions to the existing literature. Foremost, it extends our understanding of the relationship between psychological wellbeing and education levels, providing additional evidence in this domain. The study shows a clear inverse association between educational attainment and psychological distress. Individuals with higher qualifications, such as a degree, reported the lowest GHQ scores, indicating better mental health, whereas those with no formal qualifications exhibited the highest scores. The monotonic increase in GHQ across decreasing education levels suggests that lower educational attainment is consistently associated with poorer psychological wellbeing. Additionally, the study unveils distinct effects of financial situations on the psychological wellbeing of both men and women, contributing nuanced insights to the broader literature.

Furthermore, the research sheds light on several other direct contributors to psychological wellbeing, including gender, age, challenges in paying rent or mortgage, number of children, and marital status. The paper goes beyond isolated examinations and considers interactions between these factors to assess potential variations in confounding factors among different groups.

The data for this analysis was collected prior to the COVID-19 pandemic. Therefore, future studies could construct similar datasets using the Understanding Society survey to examine the impact of COVID-19 on psychological wellbeing. A comparative analysis could assess psychological wellbeing during the pandemic, as well as the long-term adverse effects post-pandemic.
